# Development and validation of a nomogram prediction model for cardiovascular comorbidities in COPD patients based on hypertension

**DOI:** 10.3389/fmed.2026.1766827

**Published:** 2026-03-04

**Authors:** Zhaojun Chen, Huan Li, Yuli Cai, Xuliang Chen, Deyi Zhou, Yihuan Su, Chaofeng Lin, Liangde Li, Dongjie Huang, Riken Chen, Xiaoling Wu, Zhenzhen Zheng, Mingpeng Xu

**Affiliations:** 1 The Second Affiliated Hospital of Guangdong Medical University, Zhanjiang, Guangdong, China; 2 The Second Affiliated Hospital of Guangxi Medical University, Nanning, Guangxi, China

**Keywords:** cardiovascular disease, chronic obstructive pulmonary disease, hypertension, nomogram, risk prediction

## Abstract

**Objective:**

This study aimed to examine the association between hypertension and cardiovascular comorbidities in patients with chronic obstructive pulmonary disease (COPD) and to construct a nomogram for predicting the risk of cardiovascular comorbidities in this population.

**Methods:**

This retrospective study included 1,447 patients with chronic obstructive pulmonary disease (COPD) and no pre-existing cardiovascular disease (CVD) from January 2018 to February 2022 at the Second Affiliated Hospital of Guangdong Medical University, with follow-up extending until August 2025. Patients were randomly assigned to a training cohort (*n* = 1,012) and an internal validation cohort (*n* = 435) in a 7:3 ratio. Additionally, 624 patients treated at the Affiliated Hospital of Guangdong Medical University between January 2019 and December 2019 were included as an external validation cohort. Variables with non-zero coefficients were first selected using least absolute shrinkage and selection operator (LASSO) regression and were subsequently entered into univariable and multivariable logistic regression analyses. A nomogram was then constructed based on the results of the multivariable logistic regression, and the predictive performance of the nomogram was further evaluated.

**Results:**

The predictors incorporated into the nomogram model were age, diabetes mellitus, hypertension, and edema. The nomogram demonstrated good discriminative performance, with an area under the receiver operating characteristic (ROC) curve (AUC) of 0.82 (95% CI: 0.78–0.85) in the training cohort, 0.82 (95% CI: 0.77–0.87) in the internal validation cohort, and 0.90 (95% CI: 0.87–0.93) in the external validation cohort.

**Conclusion:**

This study demonstrated that the nomogram shows good discriminative performance and can effectively estimate the risk of cardiovascular disease in patients with chronic obstructive pulmonary disease. Age, diabetes mellitus, hypertension, and edema were identified as independent predictors.

## 1 Introduction

Chronic obstructive pulmonary disease (COPD) is a chronic inflammatory airway disorder characterized by irreversible and progressively worsening airflow limitation, which markedly impairs quality of life and may even be life-threatening (1, 2). Cardiovascular disease (CVD) remains the leading cause of morbidity and mortality worldwide (3). COPD and CVD frequently coexist, and their concurrence is associated with worse clinical outcomes than either condition alone (4). CVD is the most common cause of death within 1 year following acute exacerbations of COPD (5). Moreover, CVD accounts for 42% of first hospitalizations and 44% of second hospitalizations among COPD patients, and arrhythmias are associated with a 70% in-hospital mortality rate and a 2.4-year survival. The in-hospital mortality rate reaches 31% in patients with severe COPD complicated by arrhythmias, compared with 8% in those without arrhythmias (6). The two diseases are complex and involve strong interactions between genetic and environmental factors. Despite sharing common risk factors—such as hypertension, physical inactivity, smoking, unhealthy lifestyle behaviors, declining lung function, and environmental pollution—the underlying mechanisms remain insufficiently understood, limiting effective treatment strategies (6–8). COPD patients have a significantly increased risk of developing CVD compared with the general population, potentially due to mechanisms including lung hyperinflation, hypoxemia, pulmonary hypertension, systemic inflammation, and oxidative stress (4). The presence of CVD not only increases mortality among COPD patients but also imposes a substantial healthcare burden.

Among various risk factors, hypertension is the most common and preventable contributor to cardiovascular disease—including coronary artery disease, heart failure, stroke, myocardial infarction, atrial fibrillation, and peripheral arterial disease—as well as chronic kidney disease and cognitive impairment. It is also the leading individual contributor to global mortality and disability (9). The association between blood pressure and CVD risk is graded and continuous, beginning at levels as low as 115/75 mmHg (10). Among individuals aged 40–69 years, each 20-mmHg increase in systolic blood pressure or 10-mmHg increase in diastolic blood pressure more than doubles the risk of death from stroke or ischemic heart disease (11). Conversely, a 5-mmHg reduction in systolic blood pressure can reduce stroke mortality by 14% and cardiovascular mortality by 9% (10). Although hypertension is highly prevalent among COPD patients, its quantitative association with the risk of CVD in this population has not been clearly defined (6).

Traditional risk-prediction tools often overlook COPD-specific clinical characteristics and therefore lack disease-tailored predictive capacity. Moreover, conventional diagnostic methods for CVD are costly and time-consuming (3). Thus, developing an individualized risk-prediction tool for COPD patients—particularly a nomogram integrating hypertension and other clinical indicators—may facilitate the early identification of high-risk individuals and enable targeted interventions to improve clinical outcomes.

## 2 Materials and methods

### 2.1 Data sources

This study included three datasets: a training set, an internal validation set, and an external validation set. The training and internal validation datasets were derived from 1,447 COPD patients without CVD admitted to the Second Affiliated Hospital of Guangdong Medical University between January 2018 and February 2022. All patients were followed until August 2025 and were categorized into CVD and non-CVD groups based on CVD occurrence during follow-up. Using RStudio, patients were randomly assigned to a training set (*n* = 1,012) and an internal validation set (*n* = 435) at a 7:3 ratio for model development and validation. The external validation dataset consisted of 624 COPD patients without CVD admitted to the Affiliated Hospital of Guangdong Medical University from January to December 2019. These patients were similarly followed and classified into CVD and non-CVD groups. Inclusion criteria required meeting the diagnostic criteria for COPD, whereas exclusion criteria were age < 18 years or incomplete clinical data.

This study was conducted in accordance with the Declaration of Helsinki and was approved by the Medical Ethics Committee of the Second Affiliated Hospital of Guangdong Medical University (2024–065), Affiliated Hospital of Guangdong Medical University (PJKT2025-099). Informed consent was obtained from all participants. For individuals unable to provide consent directly, informed consent was obtained from their legal guardians.

### 2.2 Diagnostic criteria

Traditional diagnostic approaches for CVD, including electrocardiograms (ECG), echocardiography, coronary angiography, stress testing, magnetic resonance imaging, and intracoronary ultrasonography (3). CVD were identified according to the International Classification of Diseases, Tenth Revision (ICD-10) codes, including cerebrovascular disease and stroke (ICD-10: I63.–I64.–), angina pectoris and ischemic heart disease (ICD-10: I20.0, I21–I22, I24–I25), excluding codes I25.3, I25.4, I25.10, and I25.19 (12). A diagnosis of COPD should be considered in any patient who complains of dyspnea, chronic cough or sputum production, a history of recurrent lower respiratory tract infections and/or a history of exposure to risk factors for the disease. Forced vital capacity (FVC) maneuver during spirometry showing the presence of a post-bronchodilator FEV_1_/FVC < 0.7 is needed to establish the diagnosis of COPD. The FEV_1_ also serves to determine the severity of airflow obstruction (13).

### 2.3 Follow-up and data collection

This study was designed as a retrospective cohort study. All participants were patients with COPD without cardiovascular disease at baseline and were included in the cohort for longitudinal follow-up. The time to endpoint events was calculated from baseline to the end of follow-up (August 2025) or to the occurrence of cardiovascular disease diagnosis, loss to follow-up, or death, whichever occurred first. The primary outcome was a composite endpoint defined as the occurrence of any newly diagnosed cardiovascular disease, with no restriction to first events. For patients who did not return for in-person follow-up visits, telephone interviews were conducted to collect information on health status and to ascertain whether any cardiovascular disease–related diagnoses had been made at other medical institutions. For the majority of patients, follow-up data were obtained through review of electronic medical records, with particular attention to documented cardiovascular disease diagnoses and related examinations, including electrocardiography, coronary angiography, magnetic resonance imaging, and ultrasonography.

Clinical variables included demographics (sex, age, systolic blood pressure, diastolic blood pressure, pulse, respiratory rate, smoking history), comorbidities (diabetes, hypertension, dyslipidemia, kidney disease, malignancy, edema), and laboratory indicators (C-reactive protein, white blood cell count, neutrophils, lymphocytes, monocytes, hemoglobin, platelets, albumin, glucose, cholesterol, triglycerides, HDL-C, LDL-C), totaling 32 variables.

### 2.4 Statistical analysis

All analyses were conducted using SPSS (version 27.0.1) and R software (version 4.4.3). A two-sided *P* < 0.05 was considered statistically significant. Continuous variables were expressed as mean ± standard deviation, and categorical variables as frequencies and percentages. Student’s *t*-test or the Wilcoxon rank-sum test was used for continuous variables, while the χ^2^ test or Fisher’s exact test was applied for categorical variables.

The least absolute shrinkage and selection operator (LASSO) method, which is suitable for the reduction in high-dimensional data. Features with nonzero coefficients in the LASSO regression model were selected (14–16). All candidate variables were first subjected to binomial logistic LASSO regression, with continuous variables automatically standardized prior to penalization. Ten-fold cross-validation was used to identify the optimal penalty parameter. Variables with non-zero coefficients identified by the LASSO regression were subsequently entered into univariable logistic regression analyses, and factors with *P* < 0.05 were further included in the multivariable logistic regression model. The final nomogram was constructed based on the significant predictors identified from the multivariable logistic regression analysis. Model discrimination was assessed using the area under the receiver operating characteristic (ROC) curve (AUC). The AUCs, accuracy, sensitivity, and specificity of the models were systematically compared. Calibration curves and decision curve analysis (DCA) were applied to evaluate the calibration and clinical usefulness of the models.

## 3 Results

**FIGURE 1:**
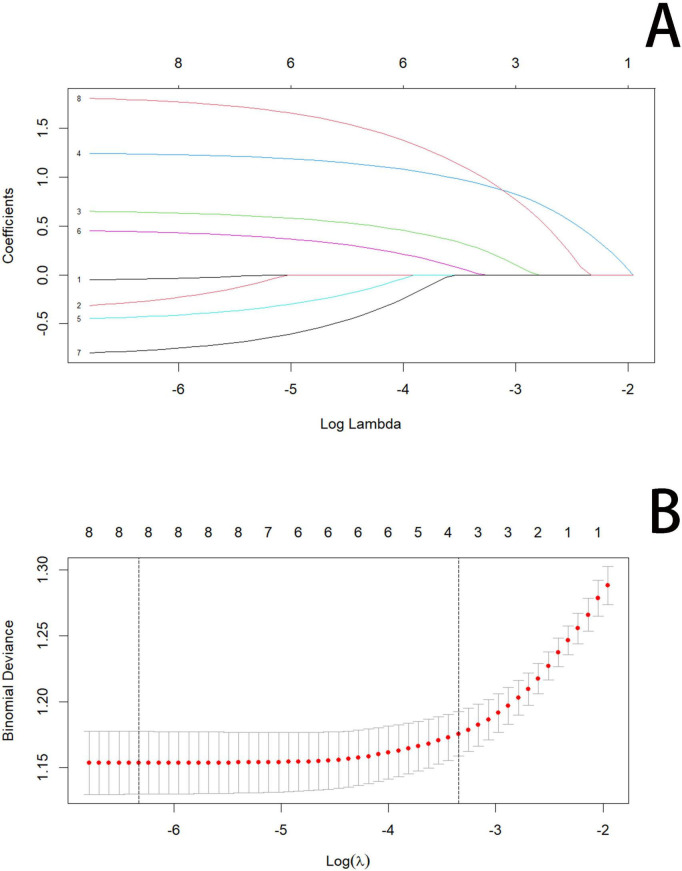
LASSO regression model. **(A,B)** 8-Edema, 4-hypertension, 3-diabetes mellitus, 6-age, 1-renal disease, 2-albumin, 5-platelet count, and 7-cholesterol.

The balance test between the training and internal validation cohorts is shown in Table 1, and no statistically significant differences were observed between the two groups except for respiratory rate and high-density lipoprotein cholesterol levels. These two variables were treated as noise and excluded from further modeling. The baseline characteristics of the training cohort are presented in Table 2, including 335 patients with CVD and 677 without CVD; 77.3% were male (*n* = 782) and 22.7% were female (*n* = 230), with a mean age of 77.00 ± 9.89 years. Based on data from the training cohort comprising 1,012 patients, eight candidate predictors with non-zero coefficients were identified using LASSO regression. These predictors included age, platelet count, albumin, cholesterol, diabetes mellitus, hypertension, renal disease, and edema (Figure 1). The optimal penalty parameter was identified as λ = 0.0018.

**TABLE 1:** Balance assessment between the training and internal validation cohorts.

Variables	Total (1,447)	Train (1,012)	Test (435)	Statistic	*P*
Age (years)	77.09 ± 9.77	77.00 ± 9.89	77.30 ± 9.50	*t* = -0.54	0.59
SBP(mmHg)	135.52 ± 20.33	135.06 ± 20.21	136.60 ± 20.58	*t* = -1.31	0.19
DBP(mmHg)	80.78 ± 24.37	80.87 ± 28.06	80.58 ± 12.03	*t* = 0.27	0.79
P(pulses per min)	85.31 ± 17.11	85.36 ± 18.13	85.20 ± 14.46	*t* = 0.17	0.86
R(breaths per min)	23.54 ± 4.79	23.34 ± 3.98	24.01 ± 6.259	*t* = -2.05	0.041
SII(10^9^/L)	2038.31 ± 3030.18	2018.80 ± 2933.78	2083.69 ± 3246.45	*t* = -0.36	0.72
NLR	9.27 ± 14.57	9.06 ± 12.25	9.74 ± 18.91	*t* = -0.69	0.49
PLR	252.88 ± 205.17	247.52 ± 201.63	265.34 ± 212.88	*t* = -1.48	0.14
LMR	2.58 ± 10.26	2.45 ± 7.69	2.90 ± 14.57	*t* = -0.62	0.54
PNI	44.89 ± 27.05	44.81 ± 21.06	45.10 ± 37.49	*t* = -0.15	0.88
CRP(mg/L)	14.33 ± 8.75	14.09 ± 5.95	14.89 ± 13.12	*t* = -1.23	0.22
WBC(10^9^/L)	9.15 ± 4.59	9.25 ± 4.82	8.90 ± 4.00	*t* = 1.44	0.15
N(10^9^/L)	7.18 ± 4.82	7.30 ± 5.25	6.88 ± 3.61	t = 1.79	0.073
LYM (10^9^/L)	1.38 ± 5.28	1.34 ± 4.05	1.46 ± 7.40	*t* = -0.30	0.77
MON (10^9^/L)	0.69 ± 0.97	0.70 ± 0.99	0.66 ± 0.88	*t* = 0.73	0.47
HB	127.06 ± 18.82	127.10 ± 18.55	126.98 ± 19.47	*t* = 0.11	0.91
PLT (10^9^/L)	223.98 ± 75.09	223.08 ± 71.48	226.06 ± 82.94	*t* = -0.65	0.51
ALB	38.01 ± 4.86	38.09 ± 4.67	37.83 ± 5.27	t = 0.92	0.36
GLU	6.94 ± 2.86	6.85 ± 2.69	7.14 ± 3.22	*t* = -1.65	0.10
CHOL (mmol/L)	4.54 ± 0.98	4.53 ± 0.97	4.56 ± 1.01	*t* = -0.51	0.61
TG (mmol/L)	1.13 ± 1.38	1.12 ± 1.33	1.15 ± 1.51	*t* = -0.39	0.70
HDL_C (mmol/L)	1.29 ± 0.36	1.28 ± 0.30	1.33 ± 0.46	*t* = -2.20	0.028
LDL_C (mmol/L)	2.76 ± 1.99	2.78 ± 2.33	2.72 ± 0.72	*t* = 0.78	0.43
Gender, n(%)				χ^2^ = 0.11	0.74
Male	1114 (77.0)	782 (77.3)	332 (76.3)		
Female	333 (23.0)	230 (22.7)	103 (23.7)		
Hypertension, n(%)				χ^2^ = 0.74	0.39
Yes	510(35.2)	349(34.5)	161(37.0)		
No	937(64.8)	663(65.5)	274(63.0)		
Smoke, n(%)					0.79
Yes	16(1.1)	12(1.2)	4(0.9)		
No	1431(98.9)	1000(98.8)	431(99.1)		
Cardiovascular Disease, n(%)				χ^2^ = 2.64	0.10
Yes	499(34.5)	335(33.1)	164(37.7)		
No	948(65.5)	677(66.9)	271(62.3)		
Diabetes, n(%)				χ^2^ = 1.24	0.27
Yes	139(9.6)	91(9.0)	48(11.0)		
No	1308(90.4)	921(91.0)	387(89.0)		
Hyperlipidemia, n(%)				χ^2^ = 0.28	0.63
Yes	95(6.6)	69(6.8)	26(6.0)		
No	1352(93.4)	943(93.2)	409(94.0)		
Tumor, n(%)				χ^2^ = 2.72	0.099
Yes	65(4.5)	39(3.9)	26(6.0)		
No	1382(95.5)	973(96.1)	409(94.0)		
Kidney disease, n(%)				χ^2^ = 2.07	0.15
Yes	196(13.5)	128(12.6)	68(15.6)		
No	1251(86.5)	884(87.4)	367(84.4)		
Edema, n(%)				χ^2^ = 0.052	0.82
Yes	88(6.1)	63(6.2)	25(5.7)		
No	1359(93.9)	949(93.8)	410(94.3)		

SBP, Systolic blood pressure; DBP, Diastolic blood pressure; P, Pulse; R, Respiratory rate (breaths per minute); SII, Systemic immune-inflammation index; NLR, Neutrophil–lymphocyte ratio; PLR, Platelet–lymphocyte ratio; LMR, Lymphocyte-to-monocyte ratio; PNI, Prognostic nutritional index; CRP, C-react protein; WBC, White blood cell; N, Neutrophil; LYM, Lymphocyte; MON, Monocyte; HB, Hemoglobin; PLT, Platelet; ALB, Albumin; GLU, Glucose; CHOL, Cholesterol; TG, Triglyceride; HDL_C, High density lipoprotein cholesterol; LDL_C, Low density lipoprotein cholesterol.

**TABLE 2:** Baseline characteristics of the training cohort.

Variables	Total (1,012)	No CVD (677)	CVD (335)	Statistic	*P*
Age (years)	77.00 ± 9.89	75.96 ± 10.16	79.11 ± 8.96	*t* = -5.03	< 0.001
SBP (mmHg)	135.06 ± 20.21	134.25 ± 20.03	136.69 ± 20.50	*t* = -1.79	0.074
DBP(mmHg)	80.87 ± 28.06	80.93 ± 33.29	80.75 ± 11.84	*t* = 0.12	0.90
P(pulse per min)	85.36 ± 18.13	84.96 ± 14.29	86.16 ± 24.11	*t* = -0.84	0.39
SII(10^9^/L)	2018.80 ± 2933.78	2045.34 ± 3141.45	1965.16 ± 2464.76	*t* = 0.44	0.66
NLR	9.06 ± 12.25	8.99 ± 12.96	9.21 ± 10.70	*t* = -0.28	0.78
PLR	247.52 ± 201.63	248.22 ± 209.67	246.12 ± 184.62	*t* = 0.16	0.87
LMR	2.45 ± 7.69	2.48 ± 7.79	2.39 ± 7.54	*t* = 0.18	0.86
PNI	44.81 ± 21.06	45.32 ± 22.44	43.78 ± 17.95	*t* = 1.17	0.24
CRP(mg/L)	14.09 ± 5.95	14.12 ± 6.31	14.02 ± 5.15	*t* = 0.26	0.80
WBC(10^9^/L)	9.25 ± 4.82	9.21 ± 4.49	9.34 ± 5.44	*t* = -0.38	0.70
N(10^9^/L)	7.30 ± 5.25	7.28 ± 5.42	7.35 ± 4.89	*t* = -0.20	0.84
LYM(10^9^/L)	1.34 ± 4.05	1.37 ± 4.32	1.29 ± 3.45	*t* = 0.31	0.76
MON(10^9^/L)	0.70 ± 0.99	0.72 ± 1.19	0.65 ± 0.38	*t* = 1.46	0.14
HB	127.10 ± 18.55	127.68 ± 17.51	125.93 ± 20.47	*t* = 1.34	0.18
PLT (10^9^/L)	223.08 ± 71.48	228.75 ± 74.84	211.61 ± 62.72	*t* = 3.83	< 0.001
ALB	38.09 ± 4.67	38.47 ± 4.88	37.33 ± 4.17	*t* = 3.90	< 0.001
GLU	6.85 ± 2.69	6.84 ± 2.93	6.87 ± 2.13	*t* = -0.17	0.86
CHOL (mmol/L)	4.53 ± 0.97	4.58 ± 0.97	4.42 ± 0.98	*t* = 2.50	0.013
TG	1.12 ± 1.33	1.13 ± 1.35	1.09 ± 1.29	*t* = 0.51	0.61
LDL_C (mmol/L)	2.78 ± 2.33	2.85 ± 2.80	2.65 ± 0.74	*t* = 1.69	0.092
Gender, n(%)				χ^2^ = 0.18	0.67
Male	782(77.3)	520(76.8)	262(78.2)		
Female	230(22.7)	157(23.2)	73(21.8)		
Hypertension, n(%)				χ^2^ = 83.37	< 0.001
Yes	349(34.5)	168(24.8)	181(54.0)		
No	663(65.5)	509(75.2)	154(46.0)		
Smoke, n(%)					1
Yes	12(1.2)	8(1.2)	4(1.2)		
No	1000(98.8)	669(98.8)	331(98.8)		
Diabetes, n(%)				χ^2^ = 24.92	< 0.001
Yes	91(9.0)	39(5.8)	52(15.5)		
No	921(91.0)	638(94.2)	283(84.5)		
**Variables**	**Total (1012)**	**No CVD (677)**	**CVD (335)**	**Statistic**	**P**
Hyperlipidemia, n(%)				χ^2^ = 2.82	0.092
Yes	69(6.8)	53(7.8)	16(4.8)		
No	943(93.2)	624(92.2)	319(95.2)		
Tumor, n(%)				χ^2^ = 1.40	0.24
Yes	39(3.9)	30(4.4)	9(2.7)		
No	973(96.1)	647(95.6)	326(97.3)		
Kidney disease, n(%)				χ^2^ = 5.94	0.015
Yes	128(12.6)	73(10.8)	55(16.4)		
No	884(87.4)	604(89.2)	280(83.6)		
Edema, n(%)				χ^2^ = 46.43	< 0.001
Yes	63(6.2)	17(2.5)	46(13.7)		
No	949(93.8)	660(97.5)	289(86.3)		

SBP, Systolic blood pressure; DBP, Diastolic blood pressure; P, Pulse; R, Respiratory rate (breaths per minute); SII, Systemic immune-inflammation index; NLR, Neutrophil–lymphocyte ratio; PLR, Platelet–lymphocyte ratio; LMR, Lymphocyte-to-monocyte ratio; PNI, Prognostic nutritional index; CRP, C-react protein; WBC, White blood cell; N, Neutrophil; LYM, Lymphocyte; MON, Monocyte; HB, Hemoglobin; PLT, Platelet; ALB, Albumin; GLU, Glucose; CHOL, Cholesterol; TG, Triglyceride; HDL_C, High density lipoprotein cholesterol; LDL_C, Low density lipoprotein cholesterol.

In the univariable and multivariable logistic regression analyses performed in the training cohort (Table 3), age, platelet count, albumin, diabetes mellitus, hypertension, renal disease, and edema were significantly associated with cardiovascular disease occurrence in patients with COPD in the univariable analysis. In the multivariable analysis, age (*P* = 0.0015, OR = 1.05), diabetes mellitus (*P* = 0.0027, OR = 2.34), hypertension (*P* < 0.001, OR = 3.34), and edema (*P* < 0.001, OR = 5.58) were identified as independent predictors of cardiovascular events.

**TABLE 3:** Univariate and multivariate analysis in the training cohort.

Variables	Univariate analysis					Multivariate analysis				
	β	S.E	Z	*P*	OR (95%CI)	β	S.E	Z	*P*	OR (95%CI)
Age	0.047	0.0080	5.82	< 0.001	1.05(1.03∼1.06)	0.045	0.014	3.17	0.0015	1.05(1.02∼1.08)
PLT	-0.0029	0.0011	-2.62	0.0088	0.99(0.99∼1.00)	-0.0022	0.0012	-1.64	0.10	0.99(0.99∼1.00)
ALB	-0.0044	0.015	-2.87	0.0041	0.95(0.92∼0.98)	0.011	0.029	0.39	0.69	1.01(0.96∼1.07)
CHOL	-0.11	0.074	-1.54	0.12	0.89(0.77∼1.03)	–	–	–	–	–
Diabetes	1.10	0.22	4.92	< 0.001	3.01(1.94∼4.68)	0.85	0.28	3.00	0.0027	2.34(1.35∼4.10)
Yes										
No										
Hypertension	1.27	0.14	8.99	< 0.001	3.56(2.70∼4.70)	1.21	0.15	7.89	< 0.001	3.34(2.48∼4.52)
Yes										
No										
Kidney disease	0.49	0.19	2.52	0.012	1.63(1.11∼2.37)	0.19	0.22	0.89	0.37	1.21(0.79∼1.85)
Yes										
No										
Edema	1.82	0.29	6.23	< 0.001	6.18(3.55∼11.25)	1.72	0.31	5.51	< 0.001	5.58(3.08∼10.52)
Yes										
No										

**FIGURE 2:**
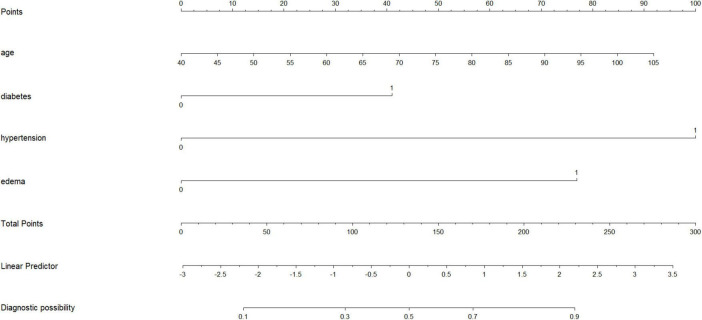
Nomogram for predicting the risk of CVD in COPD patients.

After multivariable logistic regression analysis, a nomogram for predicting the risk of cardiovascular disease in patients with COPD was constructed based on the final multivariable model (Figure 2). The nomogram incorporated four predictors, namely age, diabetes mellitus, hypertension, and edema. Using this model, the individual risk of cardiovascular disease in patients with COPD can be estimated, thereby providing a basis for targeted preventive strategies. For example, a patient aged 85 years without diabetes mellitus but with hypertension and edema would have a total score of 240 points, corresponding to an estimated 90% risk of cardiovascular disease.

**FIGURE 3:**
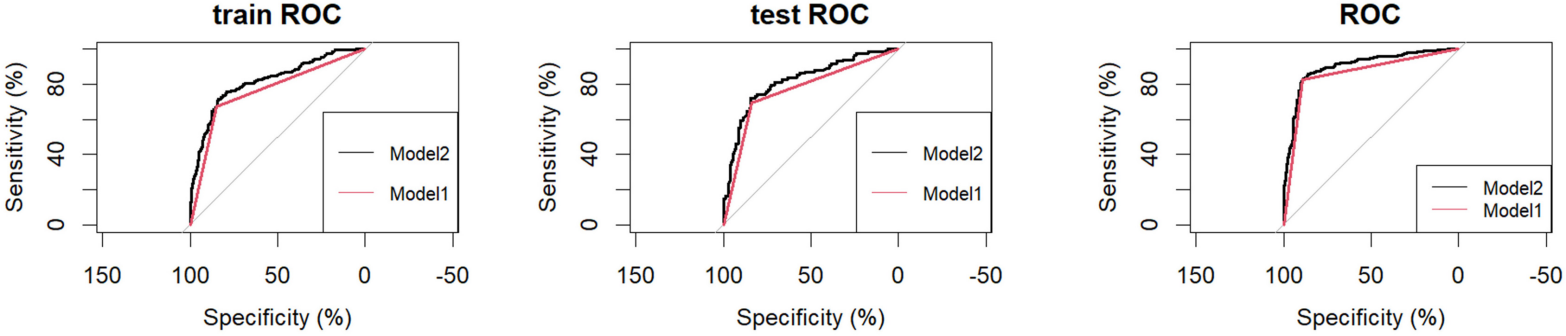
AUC of the nomogram for predicting CVD in COPD patients. Train ROC, Training cohort; Test ROC, Internal validation cohort; ROC, External validation cohort. Model1, Hypertension; Model2, Age, Diabetes, Hypertension, Edema.

**FIGURE 4:**
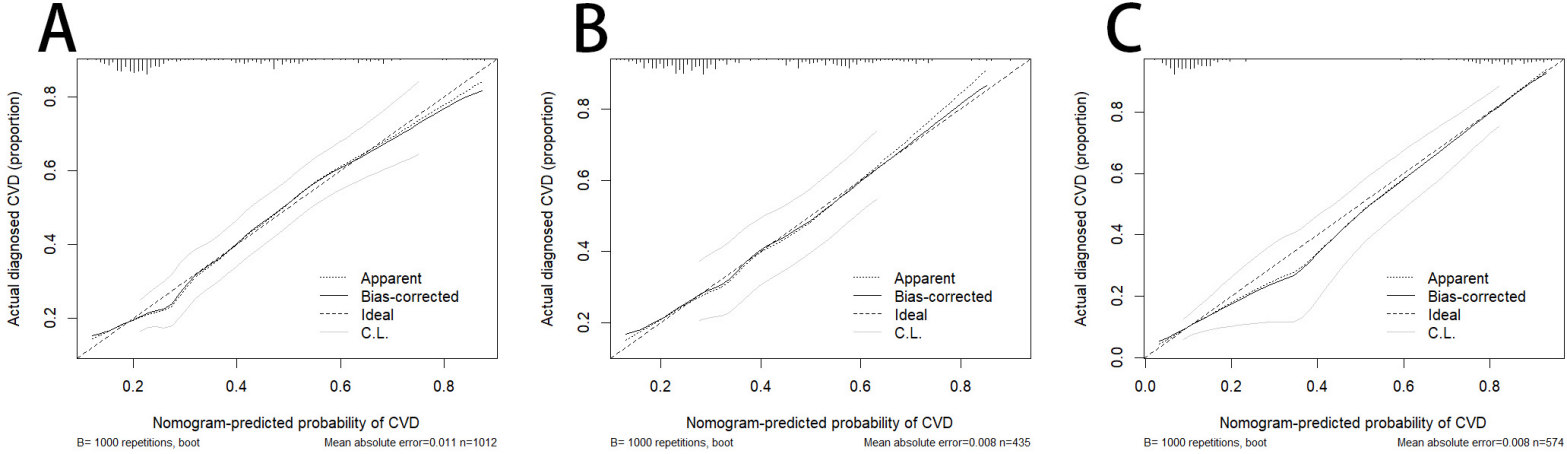
Calibration curves of the optimized nomogram for predicting the probability of cardiovascular disease in patients with COPD. **(A)** Training cohort. **(B)** Internal validation cohort. **(C)** External validation cohort.

**FIGURE 5:**
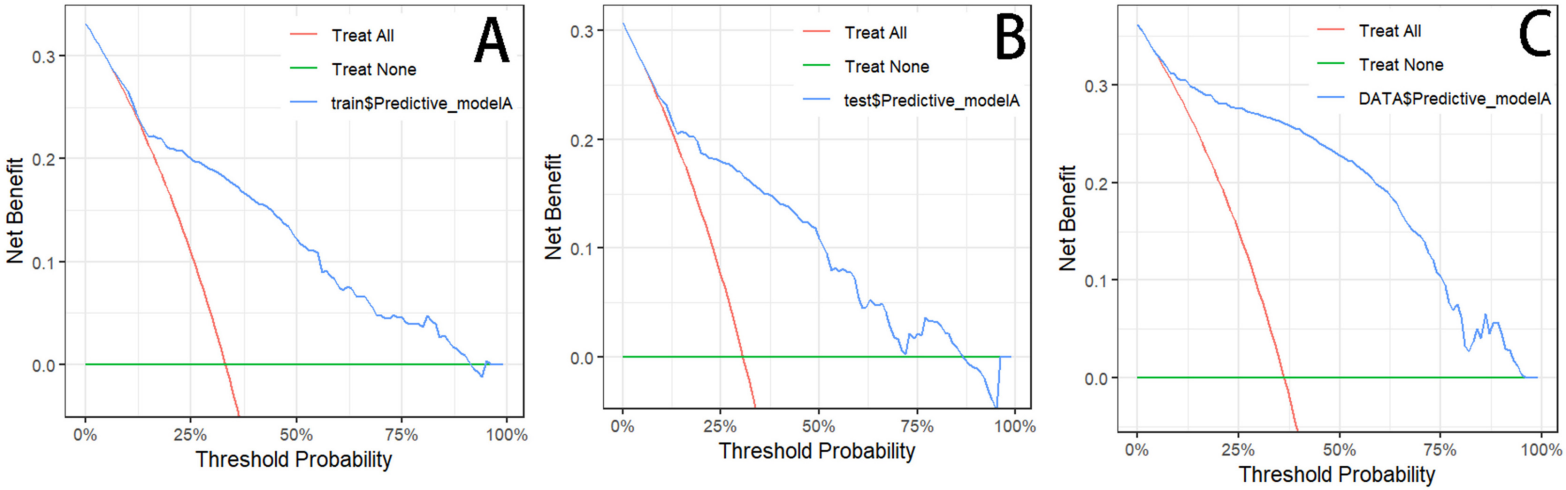
Optimized decision curve analysis of the nomogram for predicting CVD in COPD patients. **(A)** Training cohort. **(B)** Internal validation cohort. **(C)** External validation cohort.

The predictive performance of the nomogram is presented in Table 4. In the training cohort, the AUC for Model 1 and Model 2 was 0.76 (95% CI: 0.73–0.79) and 0.82 (95% CI: 0.78–0.85), respectively. Similar performance was observed in the internal validation cohort, with AUCs of 0.77 (95% CI: 0.72–0.81) and 0.82 (95% CI: 0.77–0.87). In the external validation cohort, the AUCs were 0.86 (95% CI: 0.83–0.89) for Model 1 and 0.90 (95% CI: 0.87–0.93) for Model 2. Hypertension was an effective predictor of CVD among COPD patients, and Model 2 demonstrated superior predictive accuracy, sensitivity, and specificity (Figure 3). The calibration curves of the nomogram for predicting the risk of incident cardiovascular disease in patients with COPD showed good agreement between predicted and observed probabilities in the training cohort (*P* = 0.45), internal validation cohort (*P* = 0.93), and external validation cohort (*P* = 0.52), suggesting no evidence of overfitting (Figure 4). The decision curve analysis further indicated higher net clinical benefit within the 10–90% threshold range (Figure 5).

**TABLE 4:** Predictive performance analysis of the nomogram for CVD in COPD patients.

Data	Model	AUC (95%CI)	Accuracy (95%CI)	Sensitivity (95%CI)	Specificity (95%CI)
Train	Model 1	0.76(0.73–0.79)	0.79(0.76–0.82)	0.67(0.62–0.73)	0.85(0.82–0.87)
	Model 2	0.82(0.78–0.85)	0.80(0.76–0.82)	0.72(0.67–0.79)	0.84(0.77–0.87)
Internal validation	Model 1	0.77(0.72–0.81)	0.79(0.75–0.83)	0.70(0.61–0.78)	0.84(0.79–0.88)
	Model 2	0.82(0.77–0.87)	0.81(0.74–0.85)	0.72(0.64–0.83)	0.85(0.72–0.89)
External validation	Model 1	0.86(0.83–0.89)	0.87(0.84–0.89)	0.83(0.77–0.88)	0.89(0.86–0.93)
	Model 2	0.90(0.87–0.93)	0.87(0.84–0.89)	0.83(0.78–0.89)	0.89(0.84–0.92)

Model 1, Hypertension; Model 2, Age, Diabetes, Hypertension, Edema.

## 4 Discussion

In this retrospective cohort study, we developed and externally validated a nomogram-based prediction model centered on hypertension and incorporating age, diabetes mellitus, and edema, using data from 1,447 patients with chronic obstructive pulmonary disease enrolled between 2018 and 2022 and followed up until August 2025. Multivariable logistic regression analysis demonstrated that age, diabetes mellitus, hypertension, and edema were independently associated with incident cardiovascular disease in patients with COPD. In contrast, platelet count, albumin, and renal disease, although associated with the outcome in univariable analyses, did not remain independently associated after adjustment for potential confounders. The multivariable model (Model 2) constructed from these predictors demonstrated significantly superior discriminatory performance compared with the hypertension-only model (Model 1). These findings indicate that a multi-parameter approach provides better risk stratification than a single-factor model, thereby supporting individualized risk assessment and guiding secondary prevention strategies in patients with COPD.

The findings of this study align with existing evidence regarding the roles of metabolic dysregulation, hypertension, and fluid retention in cardiovascular risk among patients with COPD. The triglyceride–glucose product index (TyG index), a reliable surrogate marker for insulin resistance, has been shown to link diabetes with cardiovascular morbidity. When the TyG index exceeds the established thresholds ( ≥ 9.18 for all-cause mortality and ≥ 9.16 for CVD mortality), it is significantly associated with increased risks of both all-cause mortality (HR 1.77, 95% CI 1.05–2.96) and cardiovascular mortality (HR 2.38, 95% CI 1.05–5.38) (17). Hypertension, another key predictor in our study, is a major contributor to cardiovascular disease and shares common pathophysiological pathways with diabetes, often clustering within the metabolic syndrome (18). Impaired lymphatic drainage may lead to peripheral edema and exert deeper cardiovascular effects by promoting interstitial fluid accumulation, systemic inflammation, and microvascular dysfunction (19). Collectively, these findings support our results indicating that diabetes, hypertension, and edema contribute substantially to cardiovascular events in the COPD population. While smoking is a well-established risk factor for both COPD and cardiovascular diseases (20, 21), previous studies have reported that even non-smokers with COPD exhibit a high prevalence of cardiovascular comorbidities (22). Consistent with this complexity, our study did not identify smoking as an independent predictor of incident cardiovascular events, suggesting that risk in COPD populations may be strongly driven by non-smoking-related mechanisms—such as chronic inflammation, oxidative stress, metabolic dysregulation, and comorbidity burden. Further research with more detailed exposure stratification, including pack-years and second-hand smoke exposure, is warranted to clarify the contribution of smoking to cardiovascular risk in COPD.

From a biological perspective, the comorbidity between COPD and CVD can be explained through multiple interrelated mechanisms. Metabolic dysregulation (23), persistent systemic inflammation (24), aging-related immune alterations, neutrophil dysfunction, and endothelial impairment collectively promote the progression of atherosclerosis and thrombosis (25), thereby increasing cardiovascular risk. Oxidative stress—widely implicated in cardiovascular conditions such as atherosclerosis and heart failure (26)—is recognized as a major driver of vascular injury in COPD. Inflammatory mediators and reactive oxygen species originating from the lungs can enter the systemic circulation, amplify systemic inflammation, destabilize vulnerable plaques, and ultimately trigger thrombotic events (27). Hypoxemia may further exacerbate this process by activating neutrophils within pulmonary microvasculature and causing endothelial damage, which can subsequently lead to pulmonary hypertension and right ventricular dysfunction. A comparative plasma proteomic study revealed that hypoxia elevates neutrophil granule proteins (NE, MPO, and NGAL), along with biomarkers of endothelial injury or activation (sICAM-1, sVCAM-1) and systemic inflammation (SAA, CRP) (28), reinforcing the central role of hypoxia-induced vascular injury. Hypoxia also reduces baroreflex sensitivity to transient blood pressure elevations in COPD patients, resulting in increased sympathetic nerve activity (29). Excessive sympathetic activation is a known contributor to stress-induced cardiomyopathy; notably, a landmark study in 2014 demonstrated enhanced sympathetic nerve activity in patients with Takotsubo cardiomyopathy (30, 31). Animal studies further support these findings. Kjeldsen et al. (32) showed that hypoxic rabbits fed a high-cholesterol diet exhibited more severe atherosclerosis, increased aortic cholesterol and triglyceride deposition, and visible myocardial infarct-like lesions, highlighting the synergistic impact of hypoxia and lipid metabolism on vascular pathology. In our study, hypertension emerged as the strongest independent predictor of incident CVD, consistent with its role as the most common and modifiable cardiovascular risk factor (10). Edema also demonstrated a high odds ratio, likely reflecting underlying cardiac dysfunction (e.g., right-sided heart failure or chronic cor pulmonale) or renal impairment, both of which substantially elevate cardiovascular risk. Although platelet count was associated with CVD in the univariate analysis—suggesting that platelet overactivation, together with the inflammatory milieu of COPD, may promote platelet–monocyte aggregate formation and contribute to atherothrombosis (6)—this association lost statistical significance after adjustment in the multivariate model. This suggests that its predictive effect may be mediated through comorbidities such as hypertension, diabetes rather than acting as a standalone risk factor. Overall, these mechanistic insights align with previous evidence linking COPD-related inflammation, oxidative stress, hypoxia, and thrombotic pathways to cardiovascular disease, offering important directions for future mechanistic research.

From a clinical perspective, the nomogram developed in this study offers several advantages. First, all included variables are routine clinical and laboratory indicators that can be easily obtained in outpatient and inpatient settings, facilitating broad implementation. Second, the model maintained good discrimination after external validation, suggesting acceptable generalizability. Third, decision curve analysis demonstrated a clear net benefit across a range of clinical thresholds, indicating that the tool can assist in identifying COPD patients who may benefit from intensified cardiovascular secondary prevention (e.g., stricter blood pressure control, optimization of glucose metabolism, and cardiac function assessment). Integrating the nomogram into electronic medical record systems could further enable automatic risk calculation and support individualized follow-up strategies.

Nonetheless, several limitations warrant consideration. The retrospective design carries inherent risks of information and selection bias, and partial telephone-based follow-up may have resulted in event misclassification. Although external validation was performed, the study population was derived from two hospitals in western Guangdong Province, with an older average age and a predominance of male participants, which may limit the generalizability of the findings. Some potentially important predictors—such as NT-proBNP, coronary imaging markers, lung function grading, and the treatment and control status of hypertension—were not included, which may have affected model performance. Imaging indicators, assessed using speckle-tracking echocardiography, can detect left and right ventricular strain, enabling the identification of early subclinical cardiac involvement and subtle myocardial injury; this approach has demonstrated both diagnostic and prognostic value (33, 34). Future prospective, multicenter studies incorporating more comprehensive variables are needed to further refine and validate the model.

In summary, this study identified age, hypertension, diabetes, and edema as key predictors of incident cardiovascular disease in COPD patients and developed a clinically applicable nomogram. While further validation is required, this model provides a practical tool for early cardiovascular risk stratification and individualized prevention in COPD populations.

## 5 Conclusion

In this study, we developed and externally validated a multifactorial nomogram based on 1,447 COPD patients followed up through August 2025. Age (OR = 1.05), diabetes (OR = 2.34), hypertension (OR = 3.34), and edema (OR = 5.58) were identified as independent risk factors for incident CVD in COPD patients. The final model—which included age, diabetes, hypertension and edema—demonstrated good discrimination, with AUCs of 0.82, 0.82, and 0.90 in the training, internal validation, and external validation cohorts, respectively. Decision curve analysis further indicated a clear net clinical benefit across a wide range of risk thresholds, supporting the model’s potential utility as a tool for clinical screening and individualized intervention.

## Data Availability

The raw data supporting the conclusions of this article will be made available by the authors, without undue reservation.
